# Mitochondrial genome and transcriptome analysis of five alloplasmic male-sterile lines in *Brassica juncea*

**DOI:** 10.1186/s12864-019-5721-2

**Published:** 2019-05-08

**Authors:** Zengxiang Wu, Kaining Hu, Mengjiao Yan, Liping Song, Jing Wen, Chaozhi Ma, Jinxiong Shen, Tingdong Fu, Bin Yi, Jinxing Tu

**Affiliations:** 1 National Key Laboratory of Crop Genetic Improvement, College of Plant Science and Technology, National Sub-Center of Rapeseed Improvement in Wuhan, Huazhong Agricultural University, Wuhan, 430070 China; 2 Institute of Vegetables, Wuhan Academy of Agricultural Sciences, Wuhan, 430070 China

**Keywords:** Mitochondrial genome, RNA editing, Alloplasmic male sterility, *Brassica juncea*, Male sterility gene

## Abstract

**Background:**

Alloplasmic lines, in which the nuclear genome is combined with wild cytoplasm, are often characterized by cytoplasmic male sterility (CMS), regardless of whether it was derived from sexual or somatic hybridization with wild relatives. In this study, we sequenced and analyzed the mitochondrial genomes of five such alloplasmic lines in *Brassica juncea*.

**Results:**

The assembled and annotated mitochondrial genomes of the five alloplasmic lines were found to have virtually identical gene contents. They preserved most of the ancestral mitochondrial segments, and the same candidate male sterility gene (*orf108*) was found harbored in mitotype-specific sequences. We also detected promiscuous sequences of chloroplast origin that were conserved among plants of the Brassicaceae, and found the RNA editing profiles to vary across the five mitochondrial genomes.

**Conclusions:**

On the basis of our characterization of the genetic nature of five alloplasmic mitochondrial genomes, we speculated that the putative candidate male sterility gene *orf108* may not be responsible for the CMS observed in *Brassica oxyrrhina* and *Diplotaxis catholica*. Furthermore, we propose the potential coincidence of CMS in alloplasmic lines. Our findings lay the foundation for further elucidation of male sterility gene.

**Electronic supplementary material:**

The online version of this article (10.1186/s12864-019-5721-2) contains supplementary material, which is available to authorized users.

## Background

Mitochondria play a vital role in plant development and reproduction, by supplying ATP via oxidative phosphorylation and serving as an energy source for multiple biochemical processes. As a semi-autonomous organelle, plant mitochondria possess an independent genome that is more dynamic and complex than their animal counterpart [1]. Plant mitochondrial genome has the following typical features: (1) a large divergence in genome size ranging from 66 kb in *Viscum scurruloideum* [2] to 11.3 Mb in *Silene conica* [3]; (2) a number of repeat sequences leading to inter- and intra-genome rearrangement [4, 5]; (3) sub-stoichiometric shift that contributes to the heterogeneous state of genome [6]; (4) promiscuous sequences derived from chloroplast and nuclear genome that contribute to genome expansion [7]; (5) relatively low mutation rate [8]; (6) high incidence of trans-splicing genes and RNA editing [9, 10]; (7) specific sequences of unknown origin [11]. At present, sequences of 238 plant mitochondrial genomes are available in the NCBI organelle genome database (https://www.ncbi.nlm.nih.gov/genome/organelle/) and the number is growing concomitant with the ongoing progression of sequencing technology. Plant mitochondria harbor an entire set of conserved genes, although they may also contain chimeric open reading frames (ORFs) that lead to male sterility [12].

Cytoplasmic male sterility (CMS) in plants is controlled by the mitochondrial genome, and is associated with a pollen sterility phenotype that can be suppressed or counteracted by the action of nuclear genes known as fertility-restoring genes (*Rf*) [11, 13, 14]. In addition to its wide application in hybrid production, CMS also provides a window to the world of plant mitochondrial-nuclear interactions. Many of CMS-related genes have been cloned from different crops [15–23], and CMS has been characterized with respect to a number of different features, including mitochondrial genome [6, 24–33], transcriptome [34, 35], proteome [36–38], miRNA [39–42], non-coding RNA [43, 44], circular RNA [45], and RNA editing [46]. Comparative mitochondrial genome sequencing between male sterility and maintainer line in CMS is often performed to elucidate sequence rearrangements by repeats, conduct phylogenetic analysis, and uncover candidate genes for CMS [26, 28, 29, 47]. On the basis of genome sequence, transcriptome analysis of mitochondria not only facilitates whole-genome expression analysis of protein-coding genes and specific ORFs, but also enables profiling of RNA editing, which is commonly observed in mitochondria [9, 48].

The example of CMS, described till date, mainly originates from natural mutations that follow the evolutionary path of sequence rearrangement and sub-stoichiometric shift [49]. However, this trait can also be generated via artificial sexual or somatic hybridization with wild relatives. CMS often develops when the cytoplasm, donated by wild relative, is incompatible with cultivar-derived nucleus, and the specific *Rf* gene is located within the genome of the wild relative. These types of alloplasmic lines often retain mitochondrial segments derived from the wild donor, also defined as mitotype-specific sequences (MSSs) [50], and is most often observed in wheat [51, 52] and Brassicas [53–55].

Numerous species within the plant family Brassicaceae are cultivated as vegetables or a source of oil, and often show strong heterosis. In addition to studies on the widely adopted Polima CMS and Ogura CMS, considerable research effort has focused on inter-specific hybridization or cell fusion between Brassica cultivars and wild Brassicaceae species, with the aim of generating alloplasmic male sterile lines [56]. Since the initial attempt, to hybridize *Brassica rapa* and *Diplotaxis muralis*, more than 20 alloplasmic male-sterile lines have been established till date [53–55, 57–62]. Among these, the cytoplasm of *Brassica oxyrrhina*, *Diplotaxis berthautii*, and *Diplotaxis erucoides* had earlier been incorporated into *Brassica juncea* via sexual hybridization using *Brassica camperstris* as a bridge [63, 64]. Similarly, the cytoplasms of *Diplotaxis catholica* and *Moricandia arvensis* had been introduced into *Brassica juncea* though sexual or somatic hybridization, followed by repeated backcrossing [65, 66]. Subsequently, the chlorotic phenotype of *Brassica oxyrrhina* and *Moricandia arvensis* in alloplasmic lines were rectified by protoplast fusion with a normal *Brassica juncea* line [67]. Furthermore, the *Rf* gene for *Moricandia arvensis* CMS was introgressed following the cross between a *Moricandia arvensis* monosomic addition line and *Brassica juncea*. This *Rf* gene has also be shown to restore the fertility of other three *Diplotaxis* CMS lines [68–72], and a nearly identical gene *orf108*, co-transcripted with atp1, has been identified as a candidate CMS gene [22, 73]. *Brassica oxyrrhina* CMS is also associated with the *orf108* gene that contains a number of single-nucleotide polymorphism (SNP) sites, but could not be restored by the *Rf* gene of *Moricandia arvensis* CMS [74, 75].

As a phenomenon that a single Rf gene could restore multiple alloplasmic male-sterile lines of different origin is uncommon, we hope to find whether the male-sterile genes in these mitochondrial genomes different or not. Therefore, the aim of present study was to assemble the five alloplasmic male sterility mitochondrial genomes, investigate the transcript levels of the genes from transcriptome data, and compare the RNA editing profiles of these genomes. The present work lays the foundation for further characterization of male sterility genes.

## Results

### Mitochondrial genome assembly and annotation

Mitochondrial DNA, extracted from purified mitochondria, was used to construct sequencing library for Illumina MiSeq and PacBio RSII platforms. The purified mitochondrial DNA was found to contribute to as much as 72% of the mitochondrial reads (see Additional file 1: Table S1). The filtered reads were de novo assembled into 10 to 25 contigs with an average N50 value of 64 kb. When combining the contigs assembled by Velvet from whole reads, each mitochondrial genome was assembled into only one or two contigs. Thereafter, PCR validation was undertaken to obtain a master circle of each mitochondrial genome.

A single SMRT cell run resulted in 1.5-Gb raw PacBio reads with an average length of 3.1 kb. When these raw reads were blast searched against the assembled *Diplotaxis catholica* CMS mitochondrial genome, 29.73% of raw reads were aligned (see Additional file 2: Table S2). This gave an average coverage of 2083 times. We also detected a large number of small inserts and deletions in the raw reads. Both MECAT and CANU can use PacBio raw reads to assemble de novo and correct any noisy reads. While the assembly of contigs with MECAT generated sequences of 159.5 kb and 6.7 kb in length, we obtained three sequences of 169.5, 75.6, and 23.9 kb using CANU. The former two contigs were identical to the MECAT contigs, whereas the 23.9-kb contig was a terminal repeat sequence that could assemble with the other two contigs. The SPAdes and Velvet-assembled contigs showed 99.99% sequence identity with the PacBio-assembled contigs.

The assembled genome was approximately 236 kb in size. *Brassica oxyrrhina* CMS and *Diplotaxis catholica* CMS have a regular *Brassica* mitochondrial genome size (220 kb), whereas the other three mitochondrial genomes were relatively larger (Table 1). The circular mitochondrial genome, generated using OGDRAW, is shown in Additional file 3: Figure S1. The five examined mitochondrial genomes have almost the same GC content of approximately 45% (Table 1). Using a local mitochondrial gene database, derived from published genomes in NCBI and MITOFY web-based blast hit, the five genomes were annotated to share almost identical gene content of 32 protein coding genes, 3 ribosomal RNAs, and 16 tRNAs. The only exception was the *Diplotaxis catholica* CMS line, in which the rps7 gene was missing. However, the amounts of tRNA varied across the genomes due to repeat sequences containing *trnY* and *trnM*. Six tRNAs (*trnD, trnI, trnL, trnM, trnN, and trnW*) were found to be of chloroplast origin, whereas 4 (*trnA, trnR, trnF, and trnV*) were missing and need to be imported from the nucleus. Overall, protein coding regions account for an average 15.6% of the genome, whereas introns account for 11.9% and the remainder includes intergenic sequences (Table 1).

**Table 1:** sequence feature of each mitochondrial genome

Cytoplasm	Brassica oxyrrhina	Diplotaxis berthautii	Diplotaxis catholica	*Diplotaxis erucoides*	*Moricandia arvensis*
GenBank accession	MG872825	MG872826	MG872827	MG872828	MG872829
length (Kb)	225.667	240.012	221.926	240.083	256.592
GC (%)	45.28	45.00	45.22	45.00	45.18
rRNAs	3	3	3	3	3
tRNAs	22	23	23	23	23
protein genes	32	32	31	32	32
Rate of coding gene (%)	16.41	15.46	16.50	15.45	14.46
Rate of intron region (%)	12.52	11.80	12.42	11.81	11.02
Rate of intergenic spacers (%)	71.07	72.74	71.07	72.74	74.52
Number of large repeats (≥1 kb)	0	1	1	1	1
Number of medium repeats (100–1000 bp)	22	33	20	29	21
Number of small repeats (< 100 bp)	96	119	121	115	180
Number of tandem repeats	20	21	18	23	32
Rate of repeat sequence (%)	3.74	6.09	7.04	6.13	8.53
Rate of Chloroplast sequence (%)	3.68	3.55	3.93	3.55	3.49
Rate of Nuclear TE sequence (%)	17.13	17.35	16.44	17.34	16.61

### Repeat sequence analysis

Plant mitochondrial genomes are characterized by large differences in the size of repeat sequences, which can be divided into dispersed and tandem repeats. Large dispersed repeats cause genome isomerization by recombination through crossing over, whereas medium repeats contribute to sequence rearrangement that often results in chimeric sequences [5, 76, 77]. On the basis of blast analyses, we were able to define the dispersed repeats. The content of repeat sequence in the five examined mitochondria varied from 3.74 to 8.53%, among which was an 11.0-kb repeat, responsible for sequence expansion in *Moricandia arvensis* CMS. The number of large, medium, and small repeats are listed in Table 1. With the exception of the *Brassica oxyrrhina* CMS line, at least one large repeat was detected in each of the examined sequences, three of which were found in *Diplotaxis* in direct orientation. Small repeats were considered to be the most abundant repeat-type. Tandem repeat sequences ranged from 12 to 59 bp in size and are listed in Table 1.

### Comparative analysis of mitochondrial genomes and mitotype-specific sequences (MSSs)

**Fig. 1:**
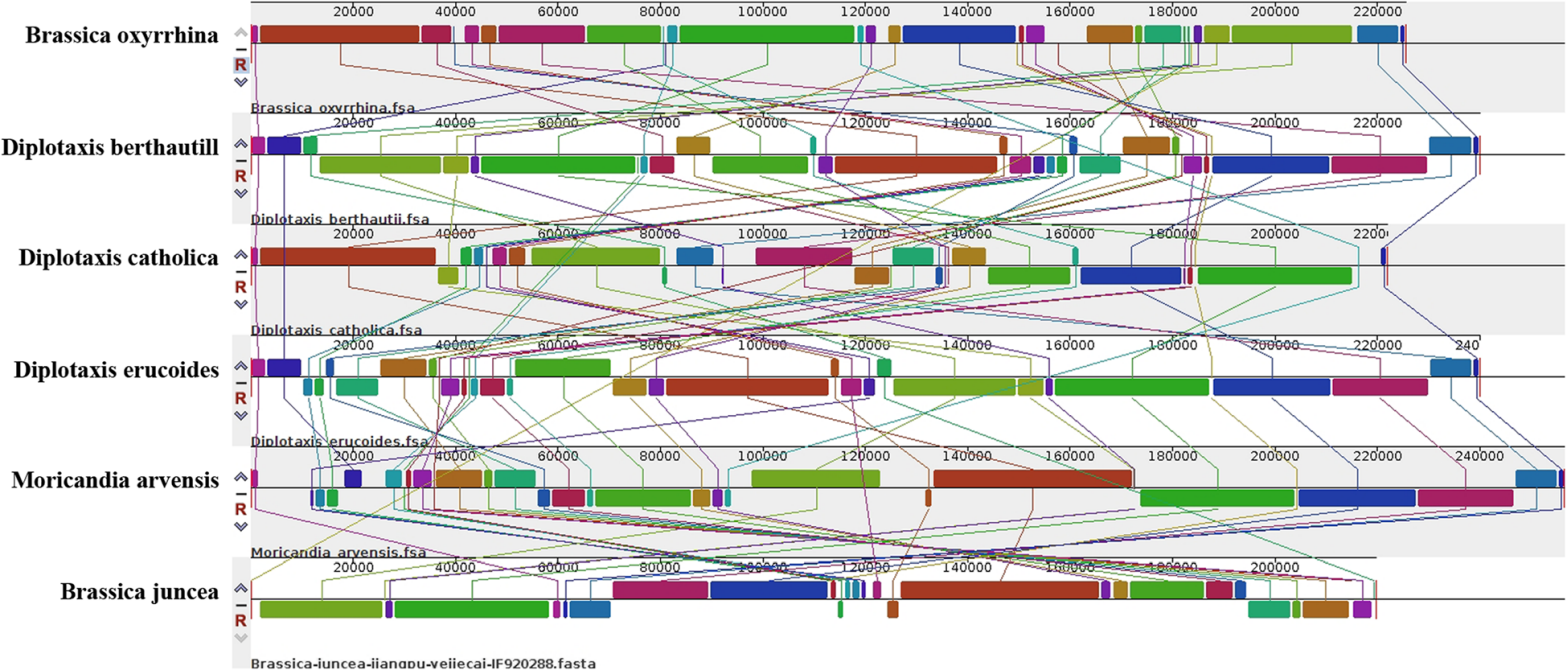
Comparative sequence analysis of alloplasmic mitochondrial genomes with *Brassica juncea* mitochondrial genome. Different blocks are assigned with different color in each mitochondrial genome, and the corresponding line that connects two blocks indicates high homology of these two blocks. Direct or reverse transcript orientation are indicated above and below the central line, respectively

Among angiosperms, species in the Brassicaceae family generally have relatively small mitochondrial genomes; Studies have shown high sequence homology among the mitochondrial genomes of plants in this family [25, 78–81]. Comparative analysis of the five mitochondrial genomes, examined in the present study with the published *Brassica juncea* var. yejiecai mitochondrial genome, revealed high sequence identity among the genomes, and identified prevalent segment inversion and arrangement (Fig. 1).

**Fig. 2:**
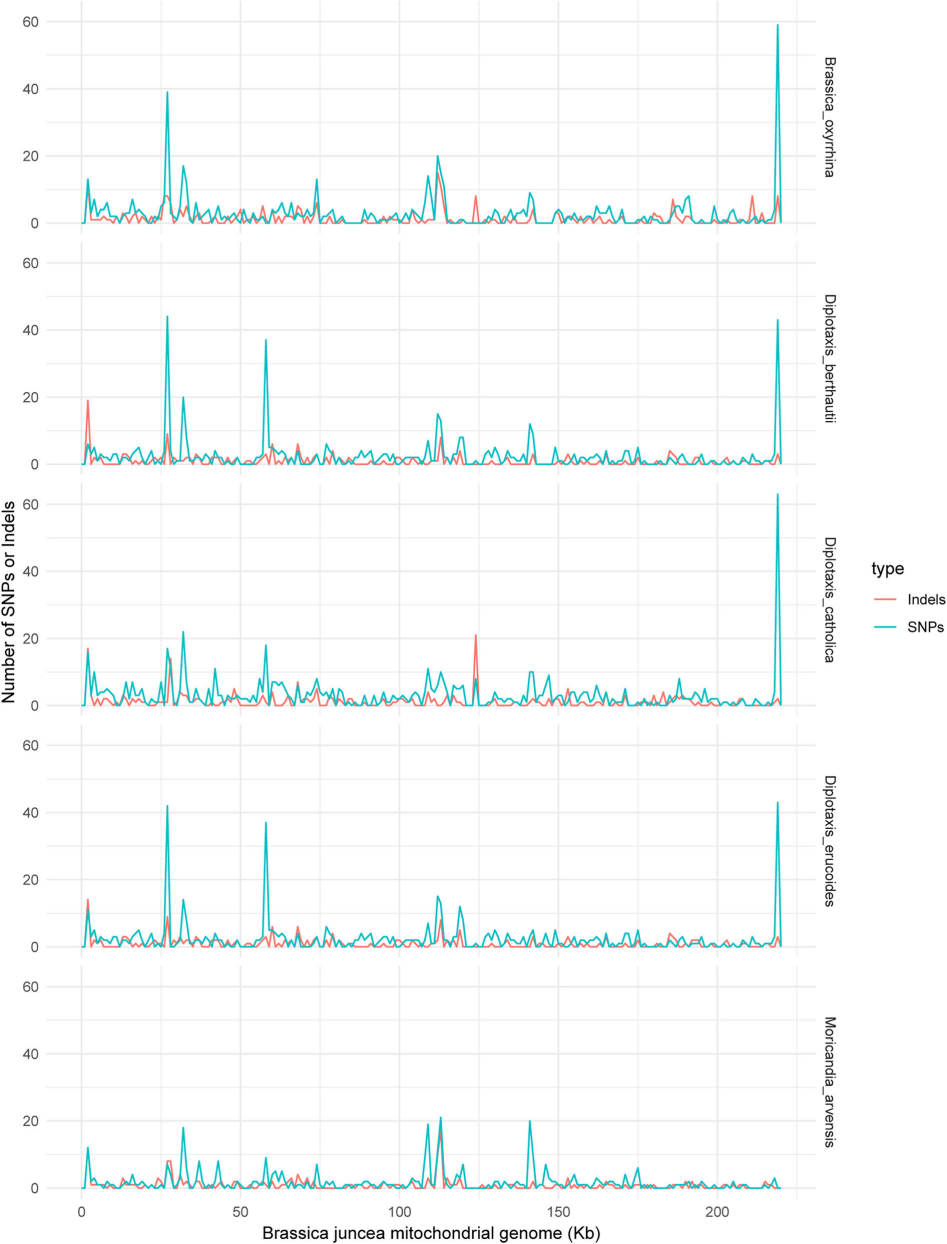
Distribution of SNPs and Indels between each alloplasmic mitochondrial genome and *Brassica juncea* mitochondrial genome. SNPs and Indels were counted from 1 kb region of *Brassica juncea* mitochondrial genome and plotted with blue and red lines, respectively

SNPs and Indels were called from GATK HaplotypeCaller by aligning reads to *Brassica juncea* mitochondrial genome; it showed difference in number and density (Fig. 2). *Diplotaxis catholica* CMS had 694 SNPs, while *Moricandia arvensis* CMS had only 389 SNPs (see Additional file 4: Table S3). About half of the SNPs were located in the gene region, and three regions were clustered with SNPs. Meanwhile, large number of Indels was also identified, and less Indels resided in the gene region. Some Indels were also clustered with SNPs and found to be a reason of mitotype-specific sequences that were aligned to homology sequences in *Brassica juncea* mitochondrial genome.

As an artificial evolutionary phenomenon, alloplasmic mitochondrial genomes contain a large number of segments derived from wild relatives, which can be defined as mitotype-specific sequences (MSSs). Using a slide window of 100-bp size with 50-bp steps, we were able to detect the MSSs of all five alloplasms, derived from the normal cytoplasm of *Brassica juncea*. The reference *Brassica juncea* mitochondrial genome includes sequences of the *Brassica juncea* hau maintainer, *Brassica juncea* var. *yejiecai*, and *Brassica juncea* var*. tumida*. The length of MSSs in *Brassica oxyrrhina* CMS and *Moricandica arvensis* CMS was 44.5 kb and 43.2 kb, respectively, whereas that in the other three averaged to 37.2 kb. Most of these MSSs segments contained or were adjacent to repeat sequences, which implied a possible integration, through repeats, during the coexistence of the two different mitotypes.

The observed MSSs sequence contained little protein-coding gene, except ORFs that mainly had no transcripts. Certain unique ORFs in MSSs have been reported be related to male sterility gene [15]. In this regard, some ORFs in MSSs, located adjacent to coding genes are specially transcribed, especially for the candidate male sterility gene *orf108* (which forms a chimeric transcript with *atp1*) and is maintained in all five mitochondrial genomes examined in the present study. We also examined other alloplasmic CMS lines and found that the male sterility gene *orf288* for *hau*-type and *orf138* for *Ogura*-type male sterility are both located in MSSs.

### Promiscuous sequences

Plant mitochondrial DNA is well documented to incorporate multiple foreign sequences from either chloroplast or nuclear genome, also referred to as promiscuous sequences [7]. In this study, the five alloplasmic lines contained sequences derived from chloroplast DNA, with an average length of 8601 bp (3.64%), ranging from 42 bp to 2186 bp, which harbor five non-redundant tRNA. In addition, we also detected partial sequences of the chloroplast-derived *psaA, psaB, rbcL, rpoB, ycf1*, and *ycf2* genes and an intact *ycf15* gene. When we blast-searched these chloroplast-derived sequences against published Brassicaceae mitochondrial genomes, we found all of them maintained in *Brassica rapa, Brassica oleracea, Brassica nigra, Brassica napus, Brassica juncea, Brassica carinata, Raphanus stivus subspecies, Eruca vesicaria,* and *Sinapis arvensis*. However, it was not the case in *Arabidopsis thaliana*, and *Schrenkiella parvula* only lost the 2186-bp segment thereby implying a different origin.

Most of the nucleus-derived sequences in mitochondrial genome are transposable elements (TE) sequences [82]. When we used TE sequences from *Brassica rapa* and *Brassica nigra* as a reference, we found that the five mitochondrial genomes examined in the present study have, on an average, 17% nucleus-derived promiscuous sequences. Thus, the overall promiscuous sequence content in these lines is approximately 21%.

### Phylogenetic analysis

**Fig. 3:**
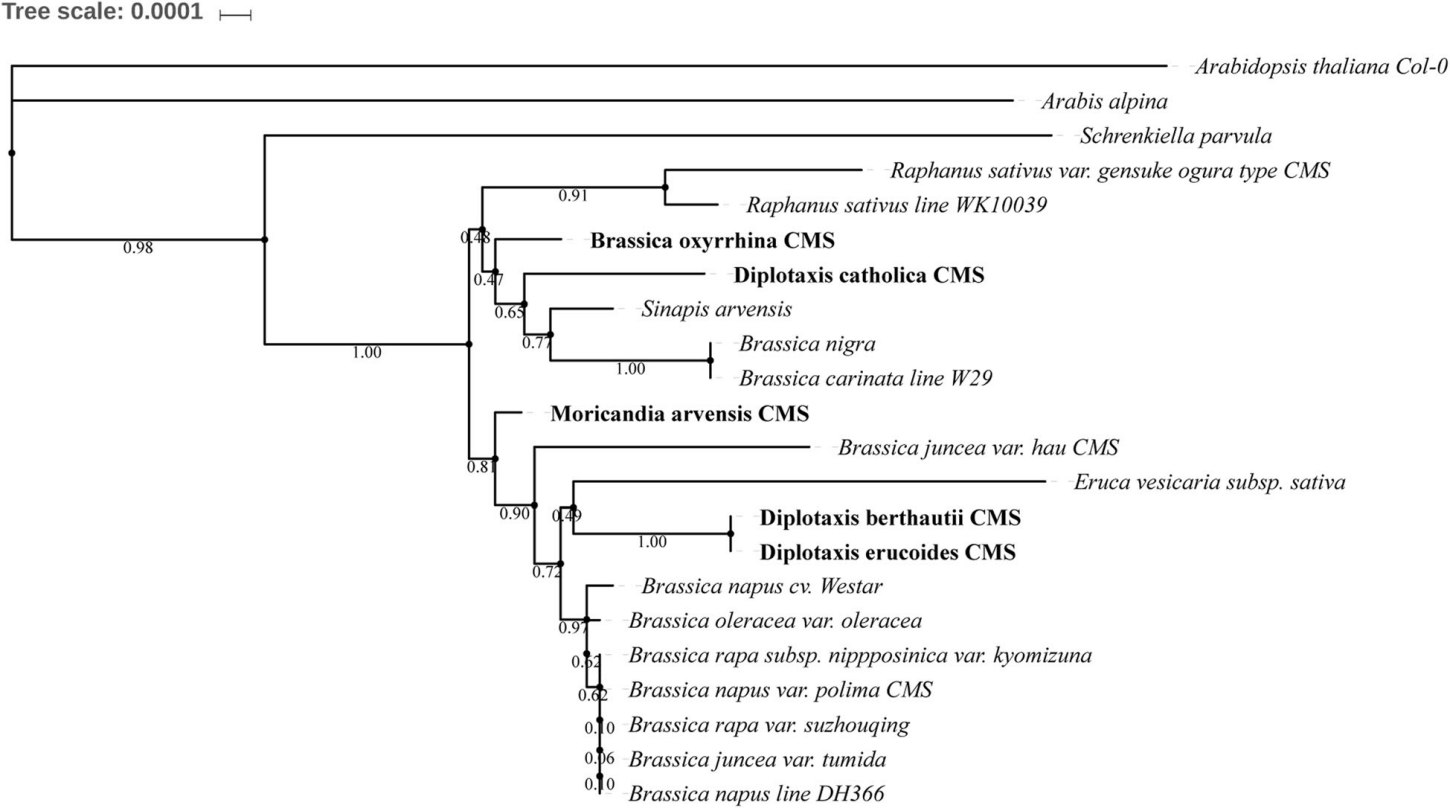
A maximum likelihood (ML) tree of sequenced alloplasmic mitochondrial genome with other Brassicaceae mitochondrial genome. Sequenced mitochondrial genomes are indicated in bold. Consensus value of each node is indicated near the fork

A maximum likelihood (ML) tree was constructed based on the concatenated sequences of 23 conserved protein coding genes from 24 mitochondrial genomes, and the computed condensed tree is shown in Fig. 3. These mitochondrial genomes include those of the commonly cultivated species of *Brassica*, wild relatives of Brassicaceae, and three other cytoplasmic male-sterile lines. The tree shows that *Diplotaxis berthautii* CMS and *Diplotaxis erucoides* CMS are located in the same node, close to *Eruca vesicaria subsp. sativa*, whereas *Diplotaxis catholica* CMS evolved from a common ancestor with *Sinapis arvensis*, and is close to *Brassica oxyrrhina* CMS. The *Moricandia arvensis* CMS has an individual origin, whereas the *Brassica juncea* var. hau CMS line is also close to wild relatives while the phylogenetic position of the *Brassica napus* var. polima CMS line is consistent with that determined previously [80].

### RNA editing profile

Mitochondrial RNA undergoes multiple processes, including transcription, editing, splicing of group I and group II introns, maturation of transcript ends, degradation, and translation [83]. RNA editing modifies the DNA code at RNA level and preserves the traditional function of genes [10, 84]. Here, we identified an average of 364 RNA editing sites located in alloplasmic protein coding genes and 81 in intergenic regions (Table 2). Details of the number of RNA editing sites in each gene are presented in Additional file 5: Table S4. Most of the editing sites reside in the first and second bases of codons, which can alter the encoded amino acids. The proportion of RNA editing sites for the first:second:third base of codons is 3:6:1, with a synonymous to non-synonymous editing ratio of 1:9. This phenomenon is relatively prevalent in angiosperm plants, with a majority of editing events resulting in the conversion of a hydrophilic amino acid to a hydrophobic residue.

**Table 2:** RNA editing profile of each mitochondrial genome

Cytoplasm		Brassica oxyrrhina	Diplotaxis berthautii	Diplotaxis catholica	Diplotaxis erucoides	Moricandia arvensis
Number of edits position	Gene	372	353	371	361	364
	Intron	30	14	32	16	16
	Intergenic	117	50	129	65	48
Edit sites location	First codon	125	116	122	118	123
	Second codon	211	208	216	210	208
	Third codon	36	29	33	33	33
Amino acid change	Synonymous	42	31	38	36	36
	Nonsynonymous	330	322	333	325	328
	Hydrophilia	61	58	60	57	60
	Hydrophobic	175	171	178	173	174
	Terminator	2	2	2	2	2

RNA editing in intronic regions is important for the correct splicing of transcripts, whereas editing sites are seldom detected adjacent to tRNAs [85]. In the present study, we detected an average of 21 RNA editing sites, located in cis-intronic regions, with the *Brassica oxyrrhina* CMS and *Diplotaxis catholica* CMS lines accounting for the most. We identified certain sites that have been edited in the *Brassica oxyrrhina* CMS and *Diplotaxis catholica* CMS, such as in the intronic regions of *ccmFc*, *cox2*, *nad2*, and *rpl2*, whereas comparable editing was absent in the other alloplasmic lines. In contrast, we were unable to detect any RNA editing site in tRNA regions.

RNA editing could also result in start or stop codon in genes. Most of the mitochondrial coding gene starts with traditional ATG codon. One exception is *nad1* that has an ACG start codon, which, however, can subsequently be modified by post-transcriptional RNA. But it is not the case with *tatC*, which starts with ATT codon. Two sites, resulting in protein truncation, were found in five alloplasmic lines, which reside in the 61-bp position of *rpl16* and 546-bp position of the second exon of *ccmFc*. However, the editing site in *rpl16* appears to be different in the *Diplotaxis catholica* CMS line, in which the 37-bp position is modified to generate a termination sequence.

### Transcript level of genes and ORFs

The mitochondrial transcriptome accounts for an average of 1.46% of the whole transcriptome data. In the present study, we evaluated the expression levels of mitochondrial genes, based on transcriptome data, using a TPM method for each genome. Among the protein-coding genes, we found *atp9* to be the most highly expressed, whereas tRNAs were the least expressed. For intergenic regions, read peaks were obtained for specific ORFs, with ORFs larger than 100 codons being annotated by ORFfinder. However, based on sequence similarity, we were unable to assign any ORF to the known genes when blasted against the NCBI NR database. Combining the quantified expression levels obtained from RNA seq data with visual inspection in IGV, only four to six ORFs were found to be transcribed in each genome and most of these were located adjacent to or co-transcribed with protein-coding genes (see Additional file 6: Table S5). Among these, *orf108* was maintained in all five alloplasmic lines.

Previous studies had identified *orf108* as a candidate male sterility gene, and a transgenic line of *orf108*, with or without a mitochondrial signal peptide, could induce 50% pollen sterility in Arabidopsis [22]. In the present study, we found that the nucleotide sequence of *orf108*, in the examined lines, is identical to that reported in previous studies. However, in the *Diplotaxis catholica* CMS line, *orf108* is a pseudogene, owing to a 2-bp frame shift [22], whereas in the *Brassica oxyrrhina* CMS line, non-synonymous mutations had resulted in five amino acid changes [75]. On the genome scale, the chimeric *orf108* and *atp1* were flanked by two pairs of small repeats. Furthermore, the *orf108* gene was found to be intact in *Eruca vesicaria subsp. sativa* and *Sinapis arvensis*.

Another gene, *orf72*, identified in an alloplasmic line of *Diplotaxis muralis*, had previously been reported as a candidate male sterility gene in *Brassica oleracea*. It is located downstream of *rps7* and contains part of the *atp9* sequence [61]. Although belonging to the same *Diplotaxis* clade, the three *Diplotaxis* mitochondrial genomes, examined in the present study, also possess this chimeric *rps7-orf72* sequence; however, *orf72* tends to be a pseudogene, owing to the presence of internal termination sites. The mitochondrial genome of *Moricnadia arvensis* CMS line also had a homologous gene to orf72, but with two additional amino acids.

Despite the considerable differences in sequence, compared to that of the other four examined mitochondrial genomes, the *Brassica oxyrrhina* CMS line acquired a segment that had 92% identity with the reported male sterility genes *orf263* [86] and *orf288* [23]. However, transcriptome data indicated that these genes are not expressed.

## Discussion

### Characteristics of the sequenced mitochondrial genomes

Plants mitochondrial genomes are usually mapped as circles, but exist physically and largely as non-circular forms. Accordingly, a master circle of the mitochondrial genome might be an artificial product assembled from genome sequencing reads. However, combined with transcriptome data, a circular representation of the mitochondrial genome is sufficient to characterize the genetic nature of the mitochondrion. Here, we assembled the mitochondrial genomes of five alloplasmic *Brassica juncea* lines, in which the cytoplasm was derived from wild Brassicaceae relatives. The size of the examined mitochondrial genomes ranged from 221 kb to 256 kb, which is slightly larger than the reported typical Brassica size [80]. However, this size range is comparable with the mitochondrial genome of other wild Brassicaceae, such as *Sinapis arvensis* (240 kb). The gene content of the examined mitochondrial genomes was found to be almost identical to the typical Brassica mitochondrial genome, and the *ccmFn* gene was found to be divided into two reading frames which is a peculiarity of *Cruciferae* species. With the exception of *Brassica oxyrrhina* CMS line, at least one large repeat sequence was identified in each of the examined mitochondrial genomes. We also identified an 11-kb repeat in the *Moricandia arvensis* CMS line, although this contained no protein-coding genes.

Comparative analysis of the mitochondrial genomes revealed similar sequence arrangements and high sequence homology among these genomes. Furthermore, we detected a large number of SNPs and Indels compared to that in *Brassica juncea* mitochondrial genome. Certain SNPs and Indels were clustered together along the genome due to MSSs homology sequences. Although recombination hotspots could be deduced from sequence arrangement, these may not be accurate in the absence of a reference ancestral mitochondrial genome sequence. In addition, we also identified MSSs, which were scattered throughout the mitochondrial genome. In plants, chimeric ORFs in MSSs are typically related to CMS; for example, a chimeric *orf182* located in an MSSs had been demonstrated to be responsible for non-pollen-type abortion in Dongxiang CMS rice [15], whereas in the present study, we found that *orf288* for *hau* CMS and *orf138* for *ogura* CMS are also located in MSSs. MSSs were identified in all genomes examined in the present study, a few of which contained expressed ORFs. Moreover, we detected the candidate male sterility gene *orf108* in MSSs of all five sequenced mitochondrial genomes, thereby implying that characterizing such regions may be a productive strategy for identifying candidate male sterility genes.

Promiscuous sequences, derived from chloroplasts and nucleus, were found to be distributed throughout the five examined mitochondrial genomes. An average of 8601 bp, comprising of 15 segments derived from chloroplasts, has been conservatively maintained in mitochondrial genomes of Brassicaceae. From a phylogenetic perspective, the incorporation of this genetic material occurred after the divergence of *Schrenkiella parvula* from a common ancestor.

### The relation of mitochondrial genomes with the crossing ways

All of the five CMS lines had resulted from two patterns of crossing: sexual hybridization and protoplast fusion. Sexual hybrids are believed to inherit mitochondrial genomes only from maternal cytoplasm, whereas in somatic hybrids there is a potential for recombination between the two different mitotypes. The three *Diplotaxis* CMS lines examined in the present study were derived from sexual hybrids, whereas the other two CMS lines had undergone protoplast fusion to rectify the chlorotic phenotype. Thus, there should be a correlation between mitochondrial genome composition and the hybridization approach used.

We found that mitochondrial genomes of the *Diplotaxis berthautii* CMS and *Diplotaxis erucoides* CMS lines were highly similar, with only seven differentially located homologous sequences. The mitochondrial genome of the *Diplotaxis catholica* CMS line was found to differ considerably from that of the other two *Diplotaxis* lines, not only in its phylogenetic position, but also due to the higher SNP content compared to that in *Brassica juncea*. With a sexual hybridization origin, they were thought to maintain the primitive mitochondrial genome.

Homologous sequences of the *Brassica oxyrrhina* CMS mitochondrial genome were found to harbor more than 500 SNPs compared to that of *Brassica juncea*, and had a greater number of SNPs and Indels in the gene region. Northern blot of the initial material also showed a wild cytoplasm pattern [87]. In contrast, mitochondrial genome of the *Moricandia arvensis* CMS line had the smallest number of SNPs compared to that of *Brassica juncea*, and also had the longest MSSs. It thus appears that the *Brassica oxyrrhina* CMS and *Moricandia arvensis* CMS lines have retained much of their original mitochondrial genome during the course of protoplast fusion.

### *Orf108* may not be the male sterility gene for *Brassica oxyrrhina* CMS and *Diplotaxis catholica* CMS

A common approach, used to identify CMS candidate genes, is to search for differences in mitochondrial gene organization, and mitochondrial transcriptomes or proteomes in lines with or without *Rf* genes [12]. Searching for ORFs in MSSs of mitochondria could also be a useful strategy [15]. The candidate CMS gene *orf108* was originally isolated based on differences in expression pattern of *atp1* determined by Northern blot analysis [73].

*Orf108* occurs as a pseudogene in *Diplotaxis catholica* CMS, whereas the *Rf* gene of *Moricandia arvensis* CMS line can restore fertility. It is possible that the *Rf* gene of *Diplotaxis catholica* CMS is tightly located with that of *Moricandia arvensis* CMS, like in the case of *Rfn* and *Rfp* for *nap* CMS and *polima* CMS, respectively, which were tightly linked independent and highly homologous genes [88]. Furthermore, the *Rf* gene of *Diplotaxis catholica* CMS line shows a sporophytic mode of fertility restoration, unlike the case of *Rf* gene of *Moricandia arvensis* CMS on *Diplotaxis berthautii* CMS and *Diplotaxis erucoides* CMS, which restored in a gametophytic way [70]. Besides, *Diplotaxis catholica* CMS and *Moricandia arvensis* CMS lines differ in terms of floral morphology, since the *Diplotaxis catholica* alloplasmic line possesses a typical petaloid anther. Sexual hybridization derived *Diplotaxis catholica* alloplasmic line showed an altered transcript pattern for *atp1* in Northern analysis, whereas in somatic hybrid derived lines an altered transcript pattern was detected only for the *coxI.* [65, 89]. The latter of these two studies identified a novel candidate of another copy of *cox1*, named *cox1–2*, which is absent in our sequenced genome. Accordingly, *orf108* may not be the gene associated with male sterility in the *Diplotaxis catholica* CMS line, even though it was fully transcribed with *atp1* by inspection in IGV.

The *Brassica oxyrrhina* CMS *orf108* gene harbors 11 SNPs that cause five non-synonymous amino acid changes at sequence level when compare to the orf108 gene from *Moricandia arvensis*, and appear to be expressed as a chimeric transcript with *atp1*. However, *Rf* gene of the *Moricandia arvensis* CMS line had been demonstrated to be unable to restore fertility in *Brassica oxyrrhina* alloplasmic lines, which is consistent with the findings of previous studies [74, 75]. Visual inspection of the transcriptome of *Brassica oxyrrhina* CMS line in IGV revealed that the chimeric transcript started from a position 162-bp downstream of the start codon of *orf108*, thus resulting in an incomplete *orf108* transcript. We, therefore, speculate that *orf108* may not be the gene associated with male sterility of the *Brassica oxyrrhina* CMS line.

These alloplasmic *Brassica juncea* lines are derived from a combination of the cytoplasm of wild relatives with the same nuclear background, but different floral morphology. Thus, the candidate male sterility gene for *Brassica oxyrrhina* CMS and *Diplotaxis catholica* CMS lines still needs further investigation.

### The difference in RNA editing profile and its relation with CMS

Sequences of mitochondrial transcriptomes were extracted from whole transcriptome data in order to analyze gene expression levels and RNA editing. In the same nuclear background, although mitochondrial protein-coding genes showed no significant difference in expression level, we found that RNA editing patterns varied, not only in the total number of editing sites but also in terms of specific location within the gene, intron, and intergenic regions. For example, *cox1* gene in the *Brassica oxyrrhina* CMS and *Diplotaxis catholica* CMS lines was found to contain two identical editing sites, whereas they were absent in the other three alloplasmic male sterile lines.

Previously, the RNA editing content in *Brassica napus* cv. Westar had been determined by sequencing mitochondrial cDNA and a total of 427 C to U conversions were identified in the ORFs [46]. Compared to the *Brassica juncea* alloplasmic lines examined in the present study, the Westar variety contained approximately 60 more RNA editing sites. This may be a species-specific characteristic or a consequence of incompatibility between wild cytoplasm and nucleus of contemporary cultivars. For example, despite their identical sequences, the *atp8* gene in Westar contained three editing sites that were not present in the five lines examined in the present study.

RNA editing can also potentially contribute to CMS, since unedited forms of an ATP subunit gene has been found to induce male sterility, whereas fertility can be restored by inhibiting its expression with antisense RNA [90–93]. In the present study, the same gene was edited differently in the same nuclear background in each cytoplasm and some of the expressed ORFs also include editing sites. In future, this could be more accurately determined with reference to the corresponding male fertility lines.

### Coincidence of CMS in alloplasmic lines

CMS can result from either intraspecific mtDNA variation or from the introduction of cytoplasm from a different species (alloplasmic CMS). Naturally occurring male sterility has been widely identified in many crops, whereas alloplasmic CMS accelerates the utilization of wild germplasms and expands the options of more stable cytoplasm. However, the underlying reason behind the cytoplasm of wild relatives often leading to CMS, when combined with a substitute nucleus, remains unanswered.

Protoplast fusion is an efficient approach for generating artificial male sterile lines from somatic hybrids with wild relatives. During this process, two different mitotypes undergo homologous recombination and the resulting hybrids are characterized by aberrant stamens. For example, asymmetric cell fusion between rapeseed (*Brassica napus*) and *Kosena* radish can lead to incorporation of the male sterility-associated gene *orf125* [25]. Furthermore, accumulated transcripts of *Arabidopsis* ORFs have been detected in a *Brassica napus* CMS line characterized by a carpelloid stamen structure, which contains mitochondrial DNA mainly derived from Arabidopsis [94]. The expression level of meristem identity and homeotic genes have been shown to be modified as a consequence of nuclear–mitochondrial incompatibility [95], which is believed to be mediated in a retrograde manner and occurs in other alloplasmic lines of tobacco, wheat, carrot, and Brassica [96, 97].

In the present study, we found that most of the expressed ORFs are located adjacent to protein-coding genes and contain SNPs or Indels compared to the homologous sequences in normal cytoplasm. Some of the alloplasmic ORFs also possess the characteristics of most of the reported male sterility genes associated with spontaneously generated male sterile lines: (1) they contain segments from known and unknown sequences; (2) they belong to mitochondrial membrane proteins that incorporate transmembrane domains; and (3) they are expressed as chimeric transcripts with known mitochondrial genes, particularly ATP-subunit genes [12]. Furthermore, the corresponding *Rf* gene is often restricted in the wild cytoplasm donor since the wild relative itself is associated with male fertility. Thereby, when normal cytoplasm is replaced with the cytoplasm of wild relative during interspecific crosses, it exposes the aberrant ORFs whose expression is specifically suppressed by the corresponding *Rf* gene in the wild relative [14]. On the other hand, ectopic ORFs can also undergo homologous recombination when two kinds of mitotype meet together during somatic fusion process. Consequently, these ORFs trigger a complicated retrograde pathway that impairs stamen development. The reason why the detrimental effect of ectopic ORFs is only apparent at reproductive stage and the corresponding retrograde pathway still needs further research.

## Conclusions

In this study, we sequenced five mitochondrial genomes of alloplasmic male sterile lines and subsequently analyzed their transcriptomes. We found that the characterized genomic features were almost identical across the genomes and repeat sequences were found to be prevalent. Although comparative alignment with the sequence of *Brassica juncea* revealed similar segment arrangement and high homology, these five alloplasmic lines maintained most of their ancestral mitochondrial genome. MSSs were identified within each of the examined mitochondrial genomes and all of them contained the candidate male sterility gene *orf108*. We found that promiscuous sequences account for approximately 21% of the genome, and that chloroplast-derived sequences are conserved among Brassicaceae-family plants. The phylogenetic positions of these genomes were found close to those of wild relatives.

Gene expression levels were deduced from the transcriptome data and we found that patterns of RNA editing differed, not only in specific protein-coding genes but also in introns and intergenic regions, which is in contrast with the pattern observed in *Brassica napus*. Here, we discussed the status of the candidate male sterility gene *orf108*, which may not be the gene responsible for male sterility in the *Brassica oxyrrhina* and *Diplotaxis catholica* lines, as per our current findings. Finally, the coincidence of CMS in alloplasmic lines was proposed. The present work provides valuable insights into the genetic features of alloplasmic CMS lines and lays the foundation for more detailed studies on the identity and characteristics of the male sterility gene.

## Methods

### Plant material

*Moricandia arvensis* and *Diplotaxis catholica* male-sterile lines were derived from the somatic hybrid or sexual allopolyploid hybrid with *Brassica juncea*, followed by repeated backcrossing to identify the stable male sterility or fertility line [65, 66]. *Brassica oxyrrhina*, *Diplotaxis berthautii*, and *Diplotaxis erucoides* male-sterile lines were introduced in *Brassica juncea* by repeated backcrossing using *Brassica camperstris* as a bridge [63, 64]. The chlorotic phenotype of *Brassica oxyrrhina* and *Moricandia arvensis* alloplasmic lines were then rectified by protoplast fusion with normal *Brassica juncea* line [67]. These five alloplasmic male-sterile lines were kindly provided by Dr. Shyam Prakash, Indian Agricultural Research Institute, India. Using K7–66, a landrace of *Brassica juncea*, as recurrent parent, all five alloplasmic lines have been backcrossed for six generations in Wuhan, Hubei province and Linxia, Gansu province between 2014 and 2017.

### Mitochondrial DNA extraction and sequencing

Open pollinated seeds of *Brassica juncea* were collected from each male-sterile line. Purified mitochondria was isolated from 7-day-old etiolated seedlings using differential centrifugation and discontinuous percoll gradient (40, 28, and 15%) centrifugation [98]. Thereafter, mitochondrial DNA was extracted with QIAamp DNA Mini Kit (QIAGEN, Hilden, Germany). The integrity, quality, and concentration of DNA were analyzed using agarose gel electrophoresis, NanoDrop 2000 (Thermo Fisher Scientific, Waltham, Massachusetts, U.S.), and Qubit fluorometer (Thermo Fisher Scientific, Waltham, Massachusetts, U.S.). Sequencing libraries, with an average insert size of 350 bp, were constructed and sequenced on Illumina MiSeq platform (Illumina, San Diego, California, U.S.). For *Diplotaxis catholica* CMS, fragment was selected with an average size of 10 kb and one single molecule real time sequencing (SMRT) cell was sequenced on PacBio RSII platform using P6-C4 reagents (Pacific Biosciences, Menlo Park, CA, U.S.). All library constructions and sequencing were performed at Personal Bio Co., Ltd., Shanghai, China.

### Mitochondrial genome assembly and annotation

The Illumina raw data output was checked individually in FastQC version 0.11.5 (http://www.bioinformatics.babraham.ac.uk/projects/fastqc), and then trimmed with Trimmomatic version 0.32 [99]. Using a list of published *Brassica* mitochondrial genome as reference (see Additional file 7: Table S6), pair-end reads that aligned at least once to the reference were selected in Bowtie2 version 2.3.2 [100]. These reads were then assembled de-novo in SPAdes version 3.10.0 with “-k 21,33,55,77,99,127 -- careful” option [101]. To avoid loss of mitochondrial sequence, specific to alloplasm, the whole reads were assembled de-novo using multiple iterations of Velvet version 1.2.10 that combine different Kmer value (91, 101, 111, 121) and expected coverage value (200, 500, 1000, 2000) [102, 103]. PacBio raw reads were individually assembled using MECAT version 1.2 and CANU version 1.5 [104, 105]. By aligning the contigs from SPAdes and Velvet, each mitochondrial genome was assembled into two or three contigs. Eventually, PCR validation was performed to get a master circle mitochondrial genome. All reads were realigned to the master circle mitochondrial genome to detect any mismatch or indel. PacBio raw reads were also realigned to the assembled Diplotaxis catholica mitochondrial genome using BLASR [106], with “--minPctIdentity 70” option.

Functional genes of mitochondrial genome were annotated by blasting against a local database, derived from published *Brassica* mitochondrial genome in NCBI GenBank, whereas tRNA was identified with tRNAscan-SE [107]. Based on the standard genetic code and alternative start codon, NCBI ORFfinder (https://www.ncbi.nlm.nih.gov/orffinder/) was utilized to identify any open reading frame (ORFs) longer than 300 nucleotides. In order to ensure the exact intron position and start or stop codon of genes, the aforementioned blast result was manually checked with the web-based annotation tool MITOFY [108]. Mitochondrial genome circle map was drawn by OGDRAW [109].

### Mitochondrial genome analysis

Mitochondrial genome was self-blasted to uncover dispersed repeats with an e-value of 1× 10^− 5^. The orientation of each repeat was checked and they were classified into three groups: large (≥1 kb), medium (100–1000 bp) and small repeats (< 100 bp). Adjacent tandem repeats were identified by the web server of Tandem Repeats Finder with default settings of 2, 7, 7, and 80 representing match, mismatch, indels, and minimum alignment score, respectively (http://tandem.bu.edu/trf/trf.html) [110]. A list of *Brassica* chloroplast genomes (see Additional file 8: Table S7) were used as blast reference to detect any chloroplast derived sequence in each mitochondrial genome.

In absence of appropriate nuclear genome sequence of *Brassica juncea*, transposable element (TE) sequence of *Brassica rapa* and *Brassica nigra* were downloaded from BRAD (http://Brassicadb.org/brad/datasets/pub/Genomes/) instead. Mitochondrial genomes were blasted against those TE sequences to identify any contamination of nucleus-derived sequences.

Comparative mitochondrial genome analysis was performed in Mauve [102]. SNPs and Indels were deduced by aligning sequencing reads to the *Brassica juncea* mitochondrial genome using HaplotypeCaller in GATK [111]. The mitochondrial genome MSSs were characterized by a slide window method with 100-bp size and 50-bp step. At least two concatenated windows that could not align to *Brassica* reference genomes were defined as MSSs, which were then extracted from the genome using a local Perl script.

### Mitochondrial RNA extraction and sequencing

Young floral buds, 1–2 mm in length (pollen mother cell stage to microspore release from the tetrad) were collected from five plants, for each line, at the same time, and immediately frozen in liquid nitrogen. Samples were stored at − 80 °C before RNA extraction. Whole genomic RNA was extracted to maximize the short RNA segments in mitochondria using TRNzol (TIANGEN Biotech., Beijing, China) method in each, with three repeats. The integrity, quality, and concentration of extracted RNA were well checked as for mitochondrial DNA.

After the DNase I (Thermo Fisher Scientific, Waltham, Massachusetts, U.S.) digestion, three independent libraries for each sample were constructed following TruSeq Stranded Total RNA Library Prep Workflow, with Ribo-Zero procedure to remove ribosomal RNA (Illumina, San Diego, California, U.S.), and then sequenced on Illumina X Ten platform (Illumina, San Diego, California, U.S.). The library was constructed and sequenced in our lab.

### Mitochondrial RNA editing content

Prior to alignment, the assembled mitochondrial genome sequence was replaced by the chloroplast-derived sequence with the same amount of “N” to avoid of misalignment of chloroplast RNA reads. After trimming of adapter sequence and low quality bases with Trimmomatic version 0.32 [99], average 6.6-Gb raw reads were aligned to the revised mitochondrial genome with HISAT2 version 2.1.0 [112]. The sam output file was manipulated with Samtools [113] and Picard tools (https://broadinstitute.github.io/picard/). Multicov function in bedtools version 2.27.0 [114] was also used to count read-coverage of gene region, which was subsequently applied to quantify the expression level of each gene using transcript per million base (TPM) method. Base recalibration of aligned reads was implemented in GATK [111] to reduce the number of false positives and inaccurate base call qualities. The HaplotypeCaller tool in GATK was last used to call single nucleotide polymorphism sites (SNPs). RNA editing site was called from filtered SNPs with a minimum variant frequency of 20% that was present at least in two independent experiments. Both C to T mutation in forward strand and G to A mutation in reverse strand were included. Mapping data of RNA sequencing and DNA sequencing were manually inspected using Integrative Genomics Viewer (IGV) [115] to verify candidate RNA editing sites. Subsequent RNA editing analysis was realized using in-house Perl scripts.

### Phylogenetic analysis

To understand the phylogenetic position of these five artificial alloplasmic lines, 17 Brassicaceae mitochondrial genomes (*Arabidopsis thaliana Col-0, Arabis alpina, Brassica carinata line W29, Brassica juncea var. tumida, Brassica juncea var. hau* CMS*, Brassica napus var. polima* CMS*, Brassica napus cv. westar, Brassica napus line DH366, Brassica nigra, Brassica oleracea var. oleracea, Brassica rapa var. suzhouqing, Brassica rapa subsp. nippposinica var. kyomizuna, Eruca vesicaria subsp. sativa, Raphanus sativus line WK10039, Raphanus sativus var. gensuke ogura type* CMS*, Schrenkiella parvula,* and *Sinapis arvensis*) were downloaded from NCBI organelle genome database (https://www.ncbi.nlm.nih.gov/genome/organelle/). Phylogenetic analyses were based on nucleotide sequences of 23 conserved protein coding genes (*atp1, atp4, atp6, atp8, atp9, ccmB, ccmC, ccmFc, ccmFn, cob, cox1, cox2, cox3, matR, nad1, nad2, nad3, nad4, nad4L, nad5, nad6, nad7,* and *nad9*), which were extracted and concatenated by local Perl script. These nucleotides were aligned using MUSCLE, implemented in MEGA X [116], and subsequently modified to manually eliminate gaps and missing data. A maximum likelihood (ML) tree was built in MEGA X and bootstrap consensus was inferred from 1000 replications. The tree was finally drawn using iTOL webserver (https://itol.embl.de/) [117].

## Additional files

Additional file 1: **Table S1.** Sequencing and assembling statistical result of each mitochondrial genome. (XLSX 10 kb)

Additional file 2: **Table S2.** Statistical result of PacBio sequencing of *Diplotaxis catholica* mitochondrial genome. (XLSX 9 kb)

Additional file 3: **Figure S1.** Mitochondrial genome circle map of 5 alloplasmic male-sterile lines in *Brassica juncea*. Different classes of conserved protein coding genes were assigned with different color; inner and outer parts of the circle mean clockwise and anticlockwise transcription. (PDF 352 kb)

Additional file 4: **Table S3.** SNP number counts between alloplasmic mitochondrial genome and *Brassica juncea* mitochondrial genome. (XLSX 9 kb)

Additional file 5: **Table S4.** Gene content of each mitochondrial genome and RNA editing number of each gene. (XLSX 13 kb)

Additional file 6: **Table S5.** Expressed ORFs in each mitochondrial genome and their location description. (XLSX 9 kb)

Additional file 7: **Table S6.** Reference mitochondrial genome list of *Brassica*. (XLSX 9 kb)

Additional file 8: **Table S7.** Reference chloroplast genome list of *Brassica*. (XLSX 9 kb)

## Abbreviations

CMS: Cytoplasmic male sterility
IGV: Integrative genomics viewer
Indels: Insert and deletions
ML: Maximum likelihood
MSSs: Mitotype-specific sequences
ORFs: Open reading frames
*Rf*: Restore of fertility genes
SMRT: Single molecule real time sequencing
SNPs: Single nucleotide polymorphism site
TE: Transposable elements
TPM: Transcript per millionbase
