# Supplementary material for: Mitochondrial genome and transcriptome analysis of five alloplasmic male-sterile lines in Brassica juncea
Source: BMC Genomics. 2019 May 8;20:348. doi: 10.1186/s12864-019-5721-2 (PMC6507029; doi:10.1186/s12864-019-5721-2)
Supplement: Supplementary file 3 — Figure S1. Mitochondrial genome circle map of 5 alloplasmic male-sterile lines in Brassica juncea. Different classes of conserved protein coding genes were assigned with different color; inner and outer parts of the circle mean clockwise and anticlockwise transcription. (PDF 352 kb) [file 12864_2019_5721_MOESM3_ESM.pdf]

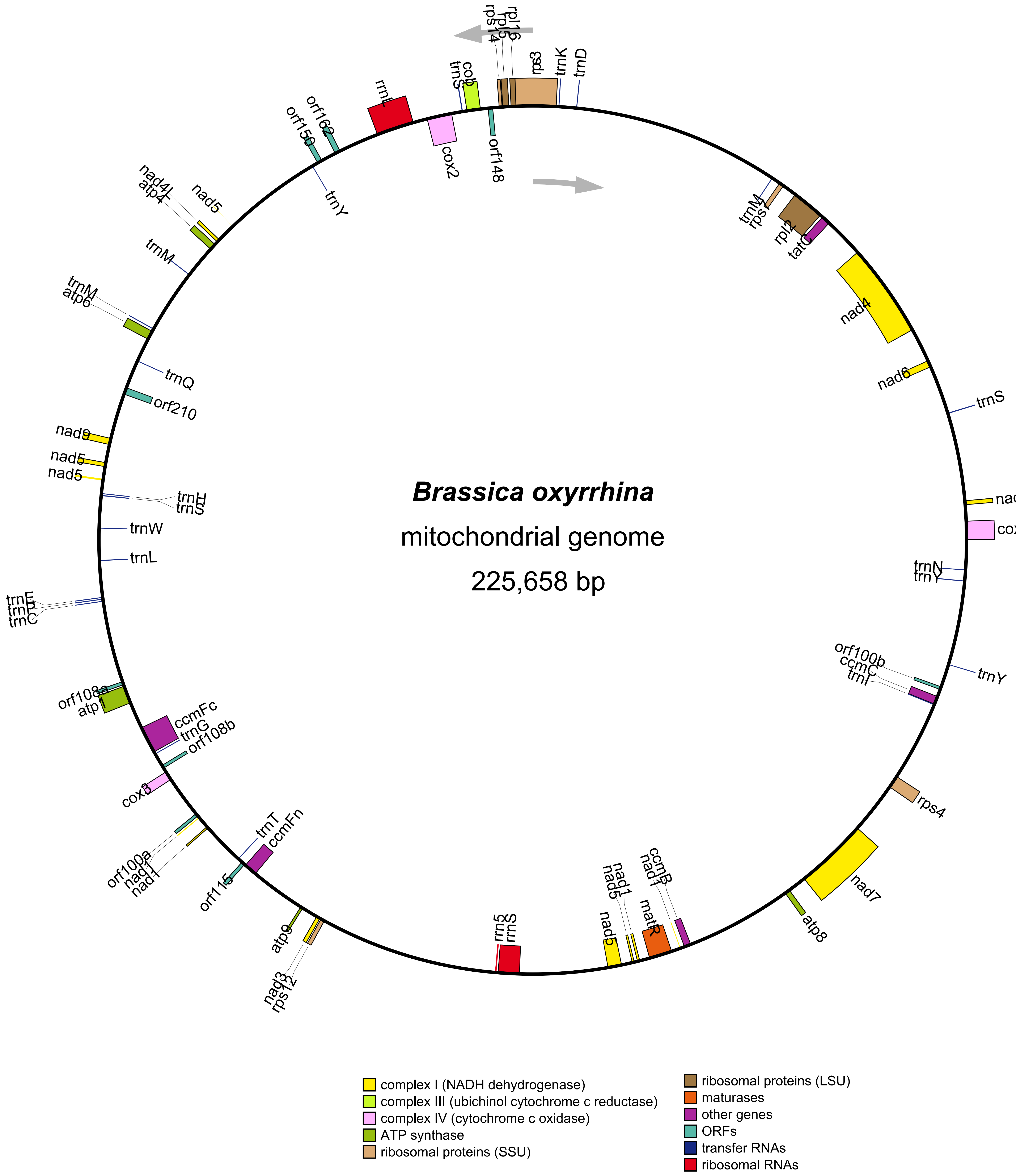

Brassica oxyrrhina
mitochondrial genome
225,658 bp
rps14
rpl5
rpl16
rps3
trnK
trnD
cob
trnS
rrnL
orf162
orf150
cox2
orf148
trnY
nad4L
atp4
nad5
trnM
atp6
trnQ
orf210
nad9
trnH
trnS
trnW
trnL
trnE
trnP
trnC
orf108a
atp1
ccmFc
trnG
orf108b
cox3
orf100a
nad1
trnT
ccmFn
orf115
atp9
nad3
rps12
rrn5
rrnS
nad5
nad1
ccmB
matR
atp8
nad7
rps4
orf100b
ccmC
trnI
trnY
trnN
cox1
nad1
trnS
nad6
nad4
rpl2
tatC
rps7
complex I (NADH dehydrogenase)
complex III (ubichinol cytochrome c reductase)
complex IV (cytochrome c oxidase)
ATP synthase
ribosomal proteins (SSU)
ribosomal proteins (LSU)
maturases
other genes
ORFs
transfer RNAs
ribosomal RNAs

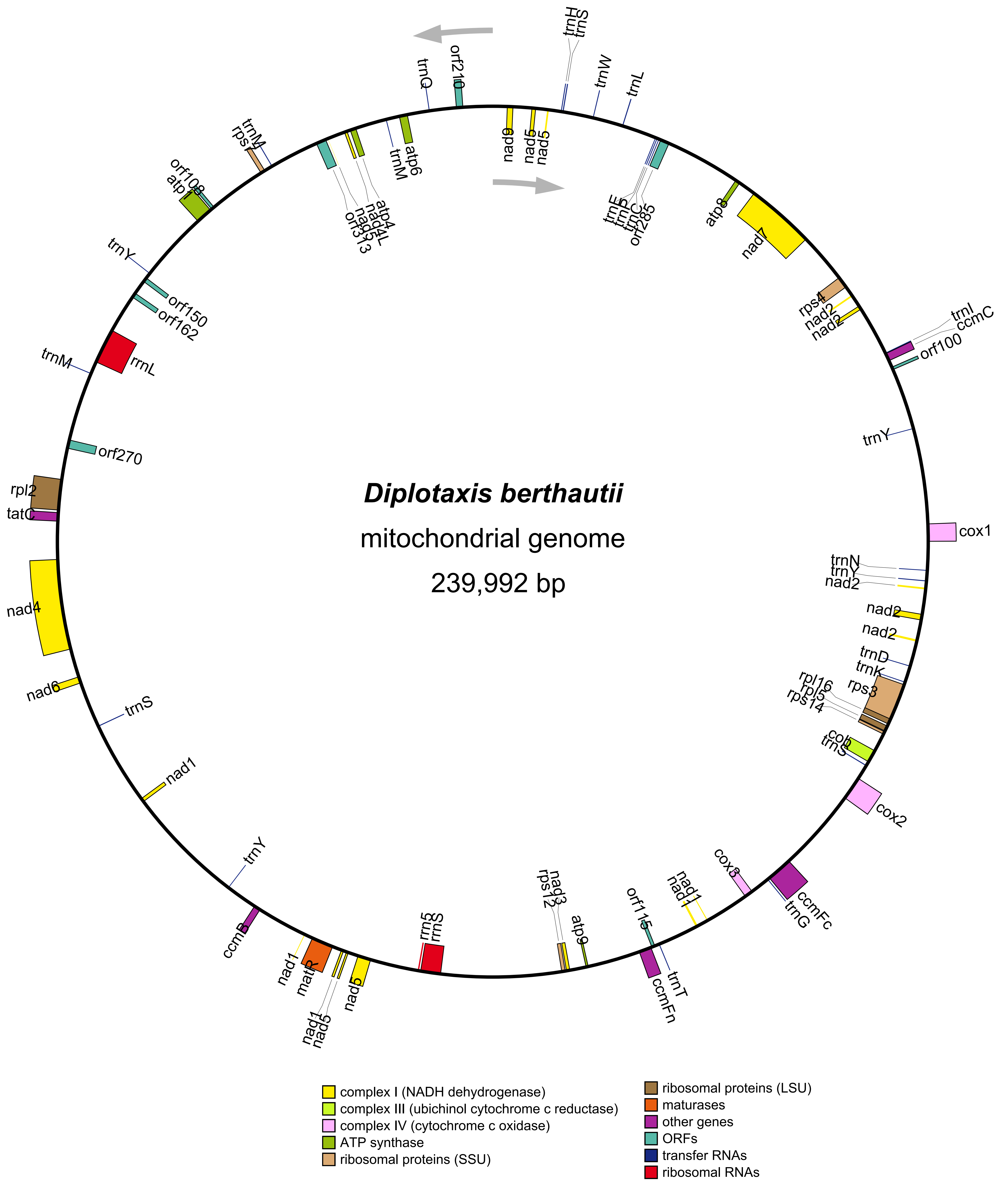
Diplotaxis berthautii
mitochondrial genome
239,992 bp
complex I (NADH dehydrogenase)
complex III (ubichinol cytochrome c reductase)
complex IV (cytochrome c oxidase)
ATP synthase
ribosomal proteins (SSU)
ribosomal proteins (LSU)
maturases
other genes
ORFs
transfer RNAs
ribosomal RNAs
trnQ
orf210
trnH
trnS
trnW
trnL
nad9
nad6
nad5
trnE
orf285
atp8
nad7
rps4
nad2
nad2
trnI
ccmC
orf100
trnY
cox1
trnN
trnY
nad2
nad2
nad2
trnD
trnK
rps3
rpl16
rpl5
rps14
cob
trnS
cox2
ccmFc
trnG
cox3
nad1
nad1
orf115
trnT
ccmFn
atp9
nad3
rps12
rrn5
rrnS
nad5
nad1
nad5
matR
nad1
ccmB
trnY
nad1
trnS
nad6
nad4
tatC
rpl2
orf270
trnM
rrnL
orf162
orf150
trnY
orf108
atp1
rps7
trnM
orf313
atp4
nad4L
trnM
atp6

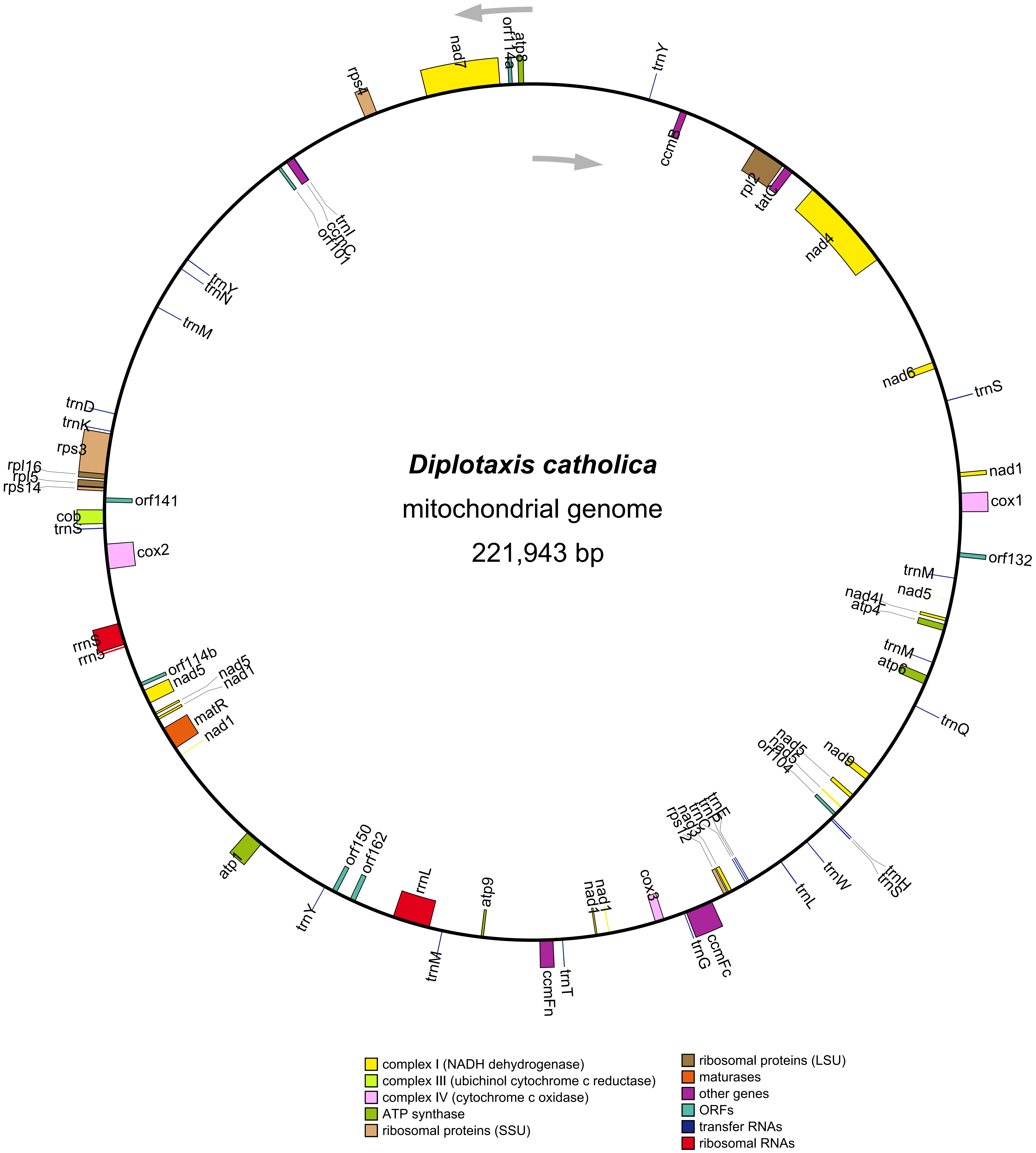

Diplotaxis catholica
mitochondrial genome
221,943 bp
atp8
orf114a
nad7
rps4
trnY
ccmB
rpl2
tatC
nad4
nad6
trnS
nad1
cox1
orf132
trnM
nad5
nad4L
atp4
trnM
atp6
trnQ
nad9
nad5
nad5
orf104
trnH
trnS
trnW
trnL
trnE
trnD
trnC
nad3
rps12
cox3
ccmFc
trnG
nad1
nad1
trnT
ccmFn
trnM
atp9
rrnL
orf162
orf150
trnY
atp1
nad1
matR
nad1
nad5
nad5
orf114b
rrn5
rrnS
cox2
trnS
cob
orf141
rps14
rpl5
rpl16
rps3
trnK
trnD
trnM
trnN
trnY
orf101
ccmC
trnI
complex I (NADH dehydrogenase)
complex III (ubichinol cytochrome c reductase)
complex IV (cytochrome c oxidase)
ATP synthase
ribosomal proteins (SSU)
ribosomal proteins (LSU)
maturases
other genes
ORFs
transfer RNAs
ribosomal RNAs

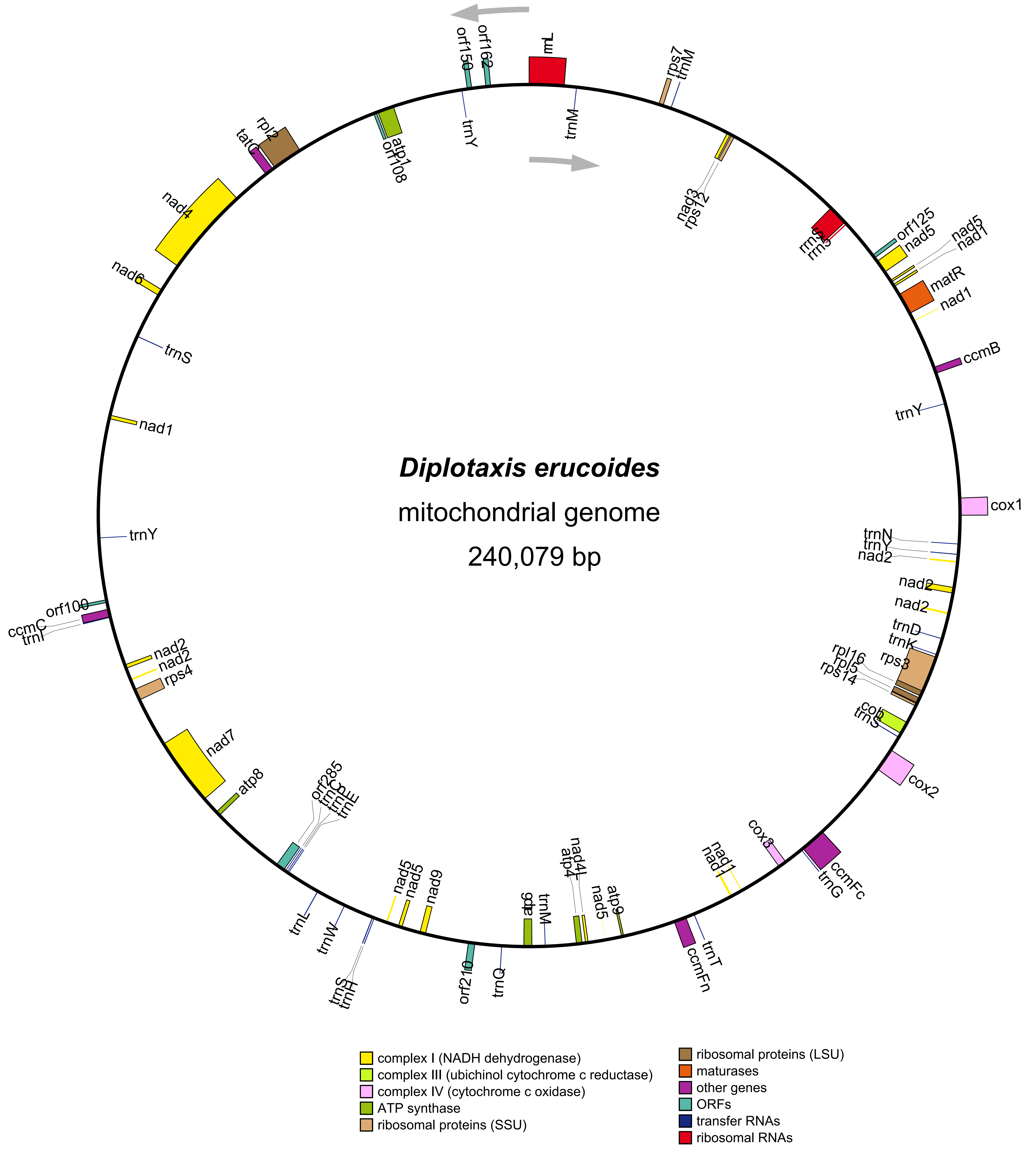

Diplotaxis erucoides
mitochondrial genome
240,079 bp
rrnL
trnM
rps7
trnM
nad3
rps12
rrn5
rrn18
orf125
nad5
nad5
nad1
matR
nad1
ccmB
trnY
cox1
trnN
trnY
nad2
nad2
nad2
trnD
trnK
rps3
rpl16
rpl5
rps14
cob
trnS
cox2
ccmFc
trnG
cox3
nad1
nad1
trnT
ccmFn
atp9
nad5
nad4L
atp4
trnM
atp6
trnQ
orf210
nad9
nad5
nad5
trnS
trnH
trnW
trnL
orf285
trnC
trnF
trnE
atp8
nad7
rps4
nad2
nad2
ccmC
trnI
orf100
trnY
nad1
trnS
nad6
nad4
tatC
rpl2
atp1
orf108
trnY
orf150
orf162
complex I (NADH dehydrogenase)
complex III (ubichinol cytochrome c reductase)
complex IV (cytochrome c oxidase)
ATP synthase
ribosomal proteins (SSU)
ribosomal proteins (LSU)
maturases
other genes
ORFs
transfer RNAs
ribosomal RNAs

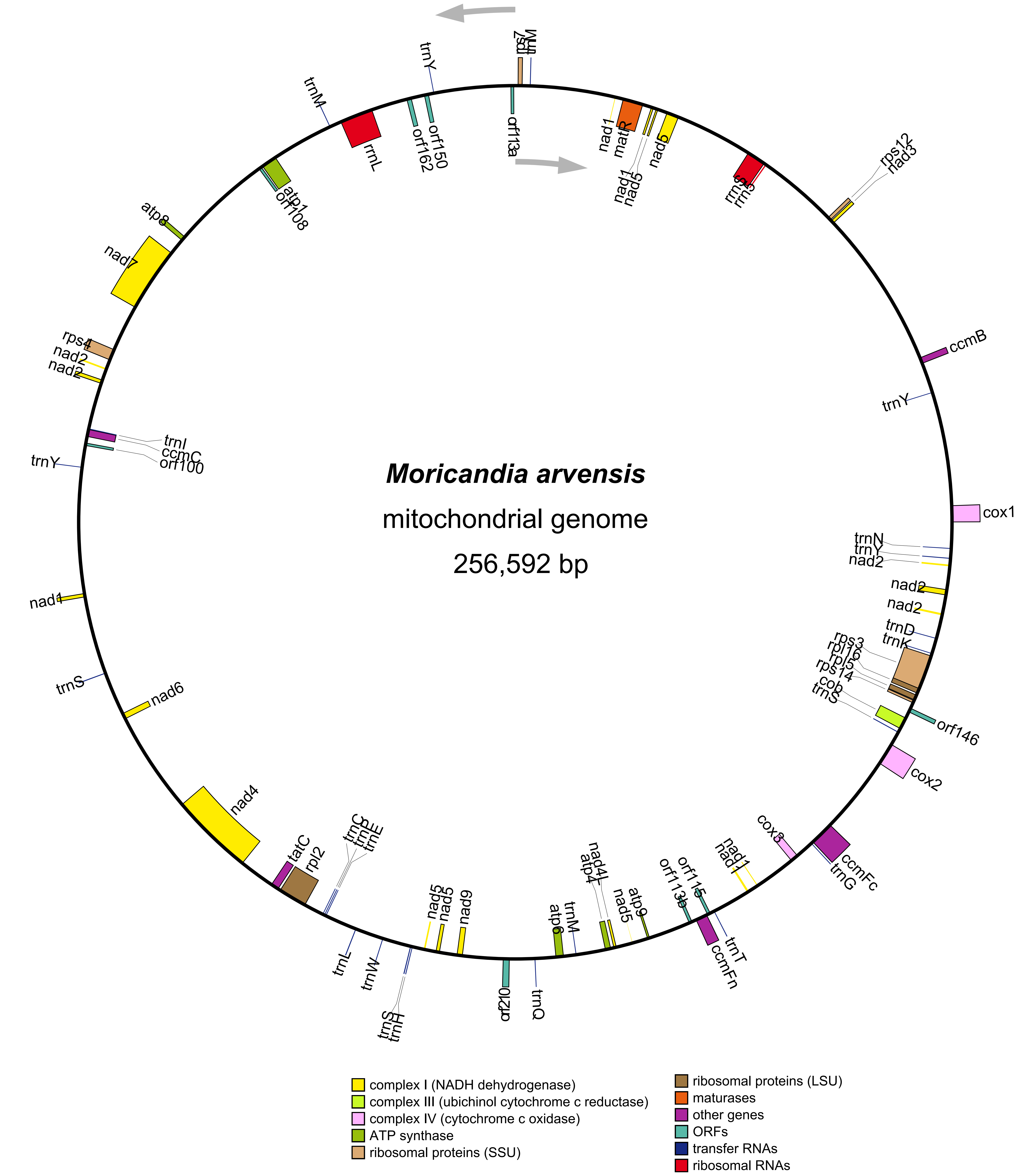
Moricandia arvensis
mitochondrial genome
256,592 bp
orf113a
rps7
trnM
trnY
orf150
orf162
trnM
rrnL
atp1
orf108
atp8
nad7
rps4
nad2
nad2
trnI
ccmC
orf100
trnY
nad1
trnS
nad6
nad4
tatC
rpl2
trnC
trnE
trnL
trnW
trnS
trnH
nad5
nad5
nad9
orf210
trnQ
atp6
trnM
atp4
nad4L
nad5
atp9
orf113b
orf115
trnT
ccmFn
nad1
nad1
cox3
ccmFc
trnG
cox2
orf146
trnS
cob
rps14
rpl5
rpl16
rps3
trnK
trnD
nad2
nad2
nad2
trnY
trnN
cox1
trnY
ccmB
rps12
nad3
rrn5
rrnS
nad5
nad1
nad1
matR
nad5
complex I (NADH dehydrogenase)
complex III (ubichinol cytochrome c reductase)
complex IV (cytochrome c oxidase)
ATP synthase
ribosomal proteins (SSU)
ribosomal proteins (LSU)
maturases
other genes
ORFs
transfer RNAs
ribosomal RNAs
